# Selenium-Binding Protein 1 Indicates Myocardial Stress and Risk for Adverse Outcome in Cardiac Surgery

**DOI:** 10.3390/nu11092005

**Published:** 2019-08-25

**Authors:** Ellen C. D. Kühn-Heid, Eike C. Kühn, Julia Ney, Sebastian Wendt, Julian Seelig, Christian Schwiebert, Waldemar B. Minich, Christian Stoppe, Lutz Schomburg

**Affiliations:** 1 Institut für Experimentelle Endokrinologie, Charité – Universitätsmedizin Berlin, corporate member of Freie Universität Berlin, Humboldt-Universität zu Berlin, and Berlin Institute of Health, D-13353 Berlin, Germany; 2 Cardiovascular Critical Care & Anesthesia Research and Evaluation (3CARE), RWTH-Aachen University, D-52074 Aachen, Germany; 3 Department of Anesthesiology, Uniklinik RWTH-Aachen, D-52074 Aachen, Germany

**Keywords:** trace element, biomarker, selenoprotein, metabolism, redox regulation, prediction

## Abstract

Selenium-binding protein 1 (SELENBP1) is an intracellular protein that has been detected in the circulation in response to myocardial infarction. Hypoxia and cardiac surgery affect selenoprotein expression and selenium (Se) status. For this reason, we decided to analyze circulating SELENBP1 concentrations in patients (*n* = 75) necessitating cardioplegia and a cardiopulmonary bypass (CPB) during the course of the cardiac surgery. Serum samples were collected at seven time-points spanning the full surgical process. SELENBP1 was quantified by a highly sensitive newly developed immunological assay. Serum concentrations of SELENBP1 increased markedly during the intervention and showed a positive association with the duration of ischemia (ρ = 0.6, *p* < 0.0001). Elevated serum SELENBP1 concentrations at 1 h after arrival at the intensive care unit (post-surgery) were predictive to identify patients at risk of adverse outcome (death, bradycardia or cerebral ischemia, “endpoint 1”; OR 29.9, CI 3.3–268.8, *p* = 0.00027). Circulating SELENBP1 during intervention (2 min after reperfusion or 15 min after weaning from the CPB) correlated positively with an established marker of myocardial infarction (CK-MB) measured after the intervention (each with ρ = 0.5, *p* < 0.0001). We concluded that serum concentrations of SELENBP1 were strongly associated with cardiac arrest and the duration of myocardial ischemia already early during surgery, thereby constituting a novel and promising quantitative marker for myocardial hypoxia, with a high potential to improve diagnostics and prediction in combination with the established clinical parameters.

## 1. Introduction

Selenium (Se) is an essential trace element affecting the expression of selenoproteins and redox signaling [1,2]. The metabolism of Se and the control of selenoprotein expression is complex as there are disease-, genotype-, sex- and potentially age-specific differences in combination with a variety of nutritional Se sources [3,4,5,6,7]. There are two major types of proteins related to Se, i.e., the selenoproteins with one or more genetically encoded selenocysteine residues in their primary sequence [8] versus the group of less-well-defined Se-binding proteins [9]. Selenium-Binding Protein 1 (SELENBP1) constitutes the most-intensively characterized member of the latter group of proteins [10]. SELENBP1 is expressed in most human and rodent tissues and migrates as a 56 kD band in electrophoretic analyses [11]. Its expression levels have been associated with tumorigenesis and cancer growth [12], especially in relation to androgen concentrations and prostate cancer [13]. A physical interaction between SELENBP1 and the major intracellular Se-dependent glutathione peroxidase (GPX1) has been described, functionally connecting both groups of Se-containing proteins [14]. Several analyses have quantified SELENBP1 in tumor tissues and report on reduced SELENBP1 expression levels in association with poor survival in gastric [15], breast [16] and renal cancer [17]. Collectively, these studies have highlighted the Se-dependent intracellular activities of SELENBP1 and a prognostic value of its expression levels in tumors as a biomarker of disease severity.

Alterations in Se blood levels and differences in the concentration of extracellular selenoproteins appear to be of similar relevance for health and disease. There are three major Se-containing circulating proteins, i.e., the plasma glutathione peroxidase (GPX3), the Se transport protein selenoprotein P (SELENOP) and selenomethionine-containing albumin. Under normal conditions, SELENOP constitutes the major fraction of extracellular Se [4], whereas the contribution of selenomethionine-containing albumin to total Se concentration in blood largely depends on the dietary intake of selenomethionine [18]. Circulating GPX3 is mainly derived from the kidneys and is regulated by hypoxia, oxidative stress and Se status [19]. Genetic inactivation of the murine *Gpx3* gene has not resulted in a major phenotype, except for effects on Se status [20]. The inactivation of *Selenop* in mice gave rise to a complex phenotype with male infertility, growth defects, oxidative stress and neurological impairment [21,22]. In patients, low SELENOP concentrations have been observed in sepsis [23], inflammatory bowel disease [24] or steatohepatitis [25] and shown to correlate to poor survival in renal cancer [26], sepsis [27] or after major trauma [28].

Information on the role and regulation of extracellular SELENBP1 is sparse. A recent report identified the protein in urine as a novel and early biomarker of acute kidney injury [29] and we described increased SELENBP1 concentrations in patients with acute coronary syndrome at high risk of major cardiac events [30]. Cardiac surgery negatively affects Se status and Se deficiency increases the risk of ischaemic heart disease [31] and promotes organ dysfunction after cardiac surgery [32]. To better characterize circulating SELENBP1 as a biomarker of myocardial stress, we monitored its concentration in patients undergoing surgery with cardioplegia-induced myocardial arrest and the use of a cardio-pulmonary bypass (CPB) and evaluated its potential diagnostic value with respect to convalescence and survival.

## 2. Materials and Methods

### 2.1. Patients

In this study, consecutive patients scheduled for elective cardiac surgery with a necessity for the use of a cardiopulmonary bypass (CPB) and cardioplegia-induced myocardial arrest were invited to participate in this analytical study. The protocol was approved by the local institutional review board (Ethics committee, RWTH Aachen University, Germany), registered at ClinicalTrials.gov (ClinicalTrials.gov identifier: NCT0126772), and all participants provided informed written consent. Adult patients scheduled for elective cardiac surgery were included, and patients who were unable to give informed consent, patients with suspicious or proven pregnancy or malignancy, and patients with perioperative infections were excluded.

### 2.2. Clinical Examination, Sample Collection and Analysis

Relevant clinical data were recorded as part of the clinical routine. Serum samples were taken from the central venous line after the induction of anaesthesia (pre-operative) 45 min after the institution of CPB (myocardial ischemia), 2 min after opening of the cross-clamps (myocardial reperfusion), 15 min after weaning from the CPB, as well as 1 h, 6 h and 24 h after admission to the ICU. Samples were stored at −80 °C until analysis. Several routine parameters were analyzed by the clinical laboratory at Uniklinik RWTH Aachen. SELENBP1 was measured as described earlier [30] at Institut für Experimentelle Endokriologie, Charité - Universitätsmedizin Berlin. Intra- and inter-assay coefficients of variation of SELENBP1 were below 10% each.

### 2.3. Statistical Analysis

All statistical analyses were performed using freely available statistical software (R 3.5.1, The R Foundation for Statistical Computing). Normal distribution of data was assessed by sample size or visual inspection of the Q-Q plot. Welch’s t-test was applied for discrete data if normality could be assumed. Wilcoxon’s rank-sum test was used if data was sparse or not normally distributed. When comparing groups of categorical data, Fisher’s exact test was applied or Chi-squared test was used for sufficiently large fields of data. Spearman’s rank correlation test was performed for testing for correlations. Friedman’s test was used when testing for changes over time due to the data not being normally distributed. Error bars for medians could not be shown, as the test assumes normality. In a post-hoc analysis, all the time-points were compared to baseline using Wilcoxon’s signed-rank test. Confidence intervals (CI) and significance were reported for an interval of 0.95 and accordingly an α of 0.05, if not stated differently. In the graphics and tables, the star symbols denote the level of significance: *: *p* < 0.05, **: *p* < 0.01, ***: *p* < 0.001, and ****: *p* < 0.0001. The following algorithm was used to determine quantiles: m = (p + 1)/3. p[k] = (k-1/3)/(n + 1/3), resulting in p[k] = median[F(x[k])]. Cross-tables and empirical receiver-operator-curves (ROC) were used to establish threshold concentrations above which the risk for adverse outcomes increased. If a threshold was found to be of diagnostic or predictive value, the area under the curve (AUC) and p-value were calculated and reported.

## 3. Results

Baseline characteristics of the patients enrolled in the clinical study are provided below. The patients underwent either a single procedure or a procedure combining two or more interventions (Table 1).

### 3.1. Preoperative Status of SELENBP1 Follows a Normal Distribution

**Figure 1:**
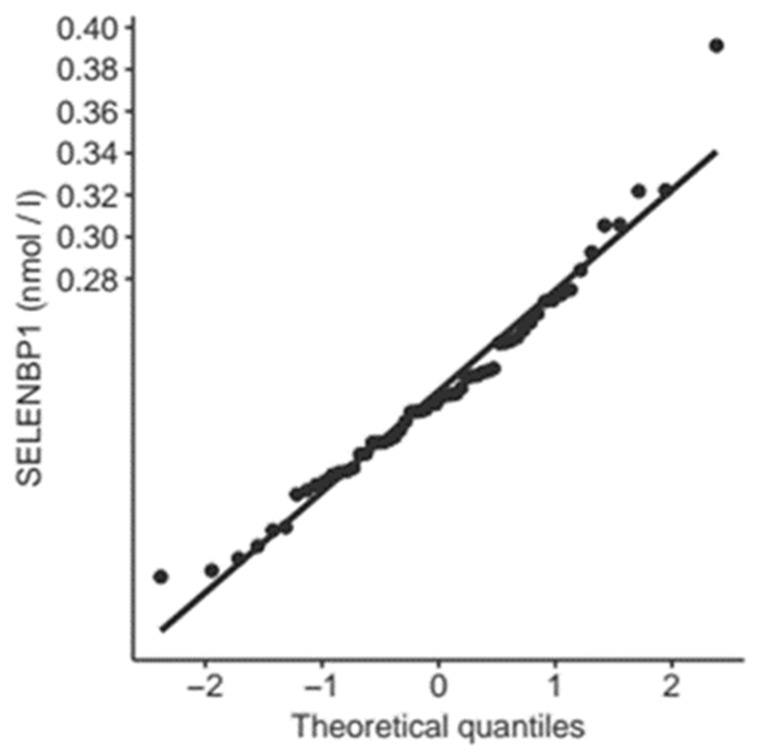
QQ-Plot of SELENBP1 in serum at baseline. The plot indicates the distribution of serum SELENBP1 concentrations at baseline before surgical intervention. The straight line indicates a normal distribution (R^2^ = 0.96, *p* < 0.0001).

The patients analyzed in this study underwent elective cardiac surgery, as opposed to an acute emergency procedure. For this reason, it was assumed that baseline serum SELENBP1 concentrations would follow a normal distribution before the intervention. Indeed, SELENBP1 values were normally distributed with the 99th percentile at 0.4 nM and the mean + 2.5 standard deviations at 0.3 nM (Figure 1), similarly to the values reported from a healthy cohort [30]. Baseline SELENBP1 concentrations were not related to perioperative risk as assessed with the EUROscore (Table 1). Two outliers were identified who started with markedly elevated baseline concentrations of circulating SELENBP1 (z = 178.2 and z = 23.6, respectively).

### 3.2. Intraoperative and Postoperative Kinetics of SELENBP1 Indicate a Transient Increase

**Figure 2:**
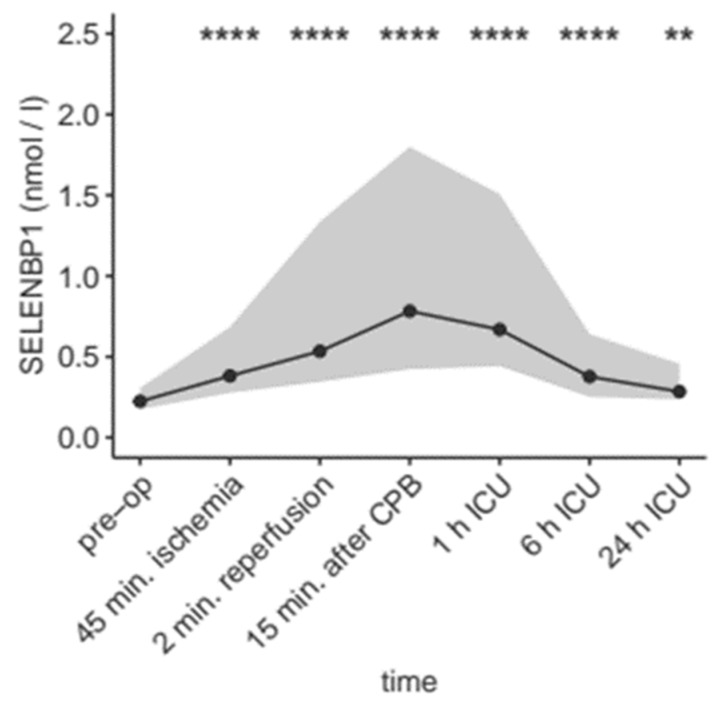
SELENBP1 before, during and after surgery. Median and 10th–90th percentile of serum SELENBP1 concentrations are provided. CPB: cardio-pulmonary bypass, ICU: intensive care unit. The values were compared to baseline by Wilcoxon’s signed-rank test. **; *p* = 0.0072, ****; *p* < 0.0001.

The blood samples taken at different time points during and after the surgical procedure were analyzed in order to assess the kinetics of circulating SELENBP1. The serum concentrations significantly increased as compared to baseline over the full course of the treatment (Figure 2).

**Figure 3:**
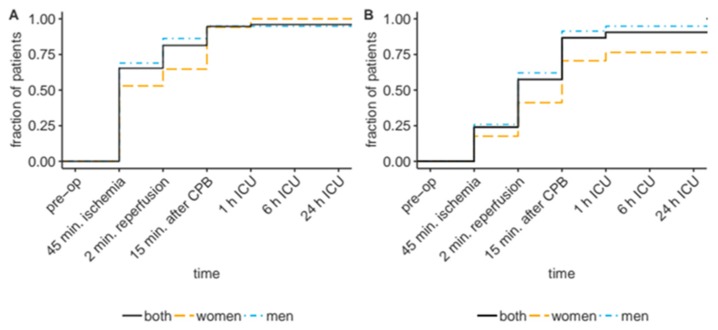
Transient SELENBP1 increased above thresholds. (**A**) Almost all the patients displayed elevated serum SELENBP1 concentrations during the intervention, as judged by an increase of at least 20% in comparison to pre-op levels, or (**B**) by surpassing a predefined cut-off value (0.455 nM), or an increase by ≥20% if the pre-op level was already elevated and >0.455 nM (empirical cumulative distribution).

During intervention, serum concentrations of SELENBP1 increased by ≥20% in n = 72 of the cardiac patients (96%, Figure 3A), and 68 patients (91%, Figure 3B) surpassed the cut-off at 0.46 nM, determined earlier as the threshold for an increased risk of death or other major adverse cardiac event in patients with suspected myocardial infarction [30]. The SELENBP1 concentrations increased until 2 min after reperfusion in 61 patients (81%), and until 15 min after weaning from the CPB in 71 patients (95%). Notably, the increase was transient, highlighting an inducing stimulus of short duration. Declining serum concentrations of SELENBP1 were observed in 65 patients (87%) within the first 6 h after admission to the ICU and in 70 patients (93%) within 24 h after admission to the ICU. In total, 74 patients (99%) showed a transient increase in serum SELENBP1 concentrations after reperfusion and qualified as responders in our hypothesis.

### 3.3. Serum SELENBP1 Concentrations Correlate to the Duration of Ischemia

Cardioplegia and the CPB exert a severe stress on the myocardium and the whole organism.

**Figure 4:**
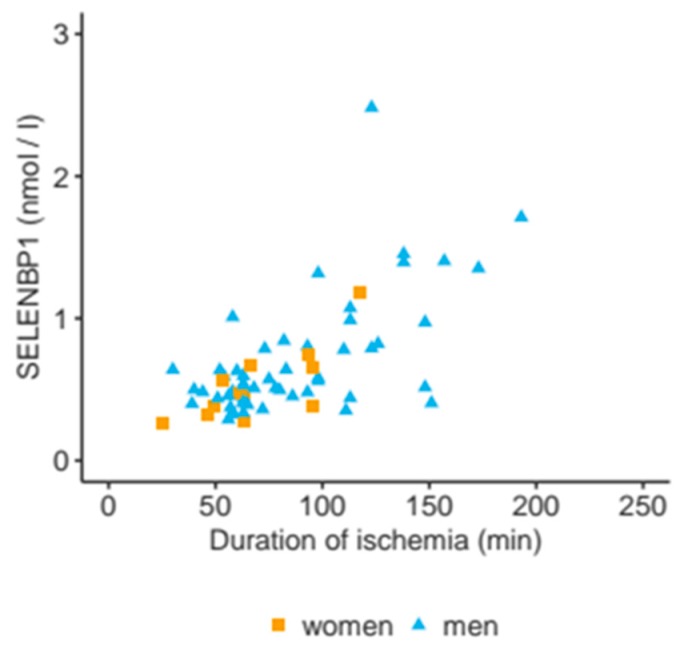
Correlation of serum SELENBP1 concentrations with duration of ischemia. Serum SELENBP1 concentrations were determined at 15 min after weaning from the CBP and are displayed in relation to duration of ischemia. A linear correlation is observed (ρ = 0.6, *p* < 0.0001).

Both interventions are related to ischemia of the heart and may constitute the major stimulus for the release of SELENBP1 into the bloodstream. Accordingly, a strong association of serum SELENBP1 concentrations with the duration of ischemia was observed already at 2 min after reperfusion (ρ = 0.5, *p* < 0.0001), and at 15 min after weaning from the CPB (ρ = 0.6, *p* < 0.0001, Figure 4). Serum SELENBP1 was slightly lower in female than in male patients at 15 min after CPB (median for female sex: 0.6 nM vs. male sex: 0.8 nM, *p* = 0.043). Fewer female patients showed a rise in SELENBP1 concentrations until 2 min after reperfusion (0.6 of female patients vs. 0.9 of male patients, *p* = 0.045), indicating a slightly lower velocity. The peak values did not differ significantly between the sexes.

### 3.4. Elevated Serum SELENBP1 Concentrations Predict Myocardial Damage

**Figure 5:**
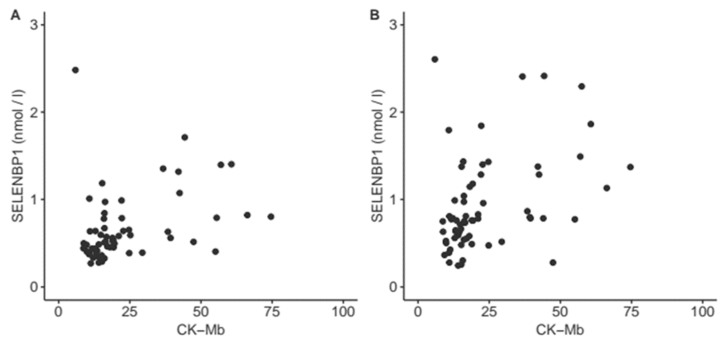
Correlation of intraoperative serum SELENBP1 concentrations with heart-specific creatinine kinase (CK-MB) after surgery. Serum SELENBP1 measured during intervention and heart-specific CK-MB concentrations measured after intervention display a positive correlation, both to the SELENBP1 concentrations measured at (**A**) 2 min after reperfusion (ρ = 0.5, *p* < 0.0001), and at (**B**) 15 min after weaning from the CBP (ρ = 0.4, *p* = 0.0007), respectively.

Circulating concentrations of SELENBP1 were unrelated to many routine laboratory markers in patients attending the emergency ward for chest pain, like troponin T, creatinine, the heart-specific isoform of creatine kinase (CK-MB), the liver enzymes ASAT or ALAT, white blood cell count (WBC), potassium or others [30]. In contrast to the former study, this analysis used samples where the time points for analysis were controlled tightly and were pre-defined, allowing a close monitoring and comparison of the kinetic behavior of cardiac biomarkers. Serum SELENBP1 concentrations increased already intraoperatively (2 min after reperfusion or 15 min after weaning from the CPB) and correlated positively to the CK-MB values measured after the intervention. This result indicates that a rise in serum SELENBP1 concentrations constituted an early event and preceded a rise in CK-MB concentrations as a marker for myocardial damage (Figure 5).

### 3.5. Elevated Serum SELENBP1 Concentrations Are Indicative of Adverse Outcomes

Next, the potential association of serum SELENBP1 with adverse outcomes was evaluated. The elevated serum concentrations of SELENBP1 were significantly associated with adverse outcomes, and predictive to discriminate patients at risk from those with negligible risk for death, bradycardia, cerebral ischemia, stay at the ICU for > 40 days, acute kidney injury or pneumonia (Table 2).

**Figure 6:**
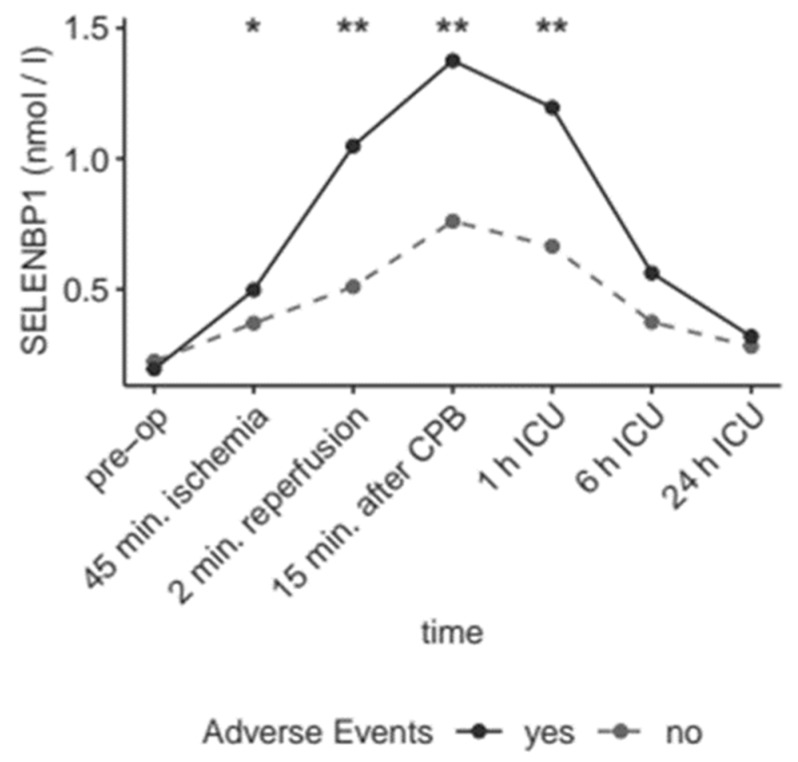
SELENBP1 before, during and after cardiac surgery in relation to outcome. Elevated serum SELENBP1 concentrations are associated with adverse events during the full course of surgery, especially directly after reperfusion, after weaning from the CPB, and shortly after arriving on the ICU. Adverse events comprise death, cerebral ischemia, bradycardia (*p* = 0.033 for 45 min after induction of ischemia, *p* = 0.0051 for 2 min after reperfusion, *p* = 0.0011 for 15 min after weaning from the CPB, and p = 0.0017 for 1 h after arrival on the ICU) (AUC; 0.8). **; *p* < 0.01, *; *p* < 0.05.

Patients suffering from bradycardia, death, or cerebral ischemia (combined endpoint 1) displayed markedly elevated serum concentrations of SELENBP1 at several points compared to those patients without any of these serious events (*p* = 0.033 at 45 min after induction of ischemia, *p* = 0.0051 at 2 min after reperfusion, *p* = 0.0011 at 15 min after weaning from the CPB, and *p* = 0.0017 at 1 h after arrival on the ICU) (Figure 6).

**Figure 7:**
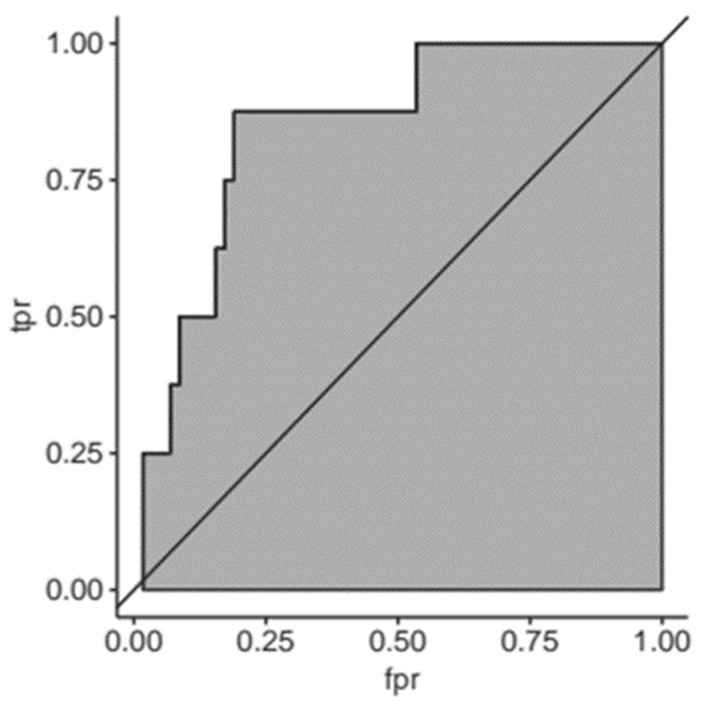
Receiver-Operator-Curve (ROC) analysis for SELENBP1 predicting adverse events. The receiver-operator-analysis for SELENBP1 concentrations and adverse events (death, cerebral ischemia, bradycardia) indicates a relatively meaningful predictive value (AUC; 0.8).

The clinical data along with the information on serum SELENBP1 were finally used to conduct a Receiver-Operator-Curve (ROC) analysis for predicting adverse events on the basis of elevated serum concentrations of SELENBP1 (Figure 7).

## 4. Discussion

In this study, circulating SELENBP1 concentrations were monitored in patients undergoing cardiac surgery along the time span from preparation for the intervention until 24 h after arriving at the ICU. The surgical intervention involved a CPB, and this procedure constitutes a clinical model for ischemia and reperfusion. Importantly, all time points could be reliably analyzed as the patients were not arriving at the hospital after an adverse incidence but underwent an elective and tightly scheduled procedure. To our surprise, a strong increase in serum SELENBP1 concentrations was detected in all but one patient. The SELENBP1 concentrations in serum reflected the myocardial damage already during surgery and nicely correlated with the levels of the heart-specific isoform of creatine kinase (CK-MB) measured after the intervention. The comparison of serum SELENBP1 concentrations with the duration of ischemia indicated an almost linear relationship, suggesting that serum SELENBP1 constituted a reliable novel marker of myocardial stress.

This notion is supported by the strong association of circulating SELENBP1 levels during and directly after the procedure, with a higher mortality risk and incidence rate of adverse events, including, e.g., length of stay at the ICU, bradycardia or cerebral ischemia. The thresholds with good positive and strong negative predictive power were in the same range as determined before in relation to chest pain and suspected myocardial infarction [30]. Collectively, serum concentrations of SELENBP1 seem to reliably indicate severe cardiac damage in patients, whereas no relevant amounts of serum SELENBP1 were detectable under control conditions in healthy subjects where SELENBP1 is located intracellularly [10,14,34]. Notably, the increased levels and fast kinetics of circulating SELENBP1 were not affected by traditional risk-factors and the baseline values did not reflect perioperative risk as assessed by the EURO-Score [33], supporting the notion that circulating SELENBP1 reflects acute events rather than indicating chronic risk-related factors. It remains to be studied whether the extent of SELENBP1 release is related to baseline Se status, as determined by the activity of circulating GPX3, SELENOP or total serum Se concentrations, and whether SELENBP1 is increased in chronic inflammatory heart disease as a potentially valuable prospective biomarker.

The previous study indicated that elevated serum concentrations of SELENBP1 in patients suspected of myocardial infarction were indicative of a higher mortality and other major adverse cardiac events [30]. There were no significant correlations with the commonly used clinical markers, such as troponin, as measured with a high-sensitivity assay, aspartate aminotransferase, creatinine, fibrinogen, prothrombin time or blood cell counts [30]. Consequently, we assumed that the stimulus for the release of SELENBP1 into the bloodstream should be different from necrosis of cardiomyocytes, which would be associated with increased circulating cardial troponins. The fast appearance of SELENBP1 already during surgery and the linear relationship between circulating SELENBP1 and the duration of myocardial ischemia suggests that SELENBP1 may have been released by cardiomyocytes upon stress and before necrosis. This hypothesis is supported by the moderate linear correlation of serum SELENBP1 during surgery to CK-MB measured at later time-points and the biologically plausible time span needed for necrosis to occur in response to hypoxia and acidosis [35].

Heart tissue is not the only potential source of extracellular SELENBP1, as urinary SELENBP1 was described as a new biomarker of heavy metal-induced kidney injury, where the protein increased strongly in the renal cortex after mercury administration in a rodent model of nephrotoxicity [36]. The authors propose urinary SELENBP1 as a novel sensitive and specific biomarker for early stages of kidney injury before irreversible damage has taken place [29,36]. Accordingly, serum SELENBP1 may indicate severe and acute noxae to cardiomyocytes at an early time point before the onset of necrosis, i.e., at a time when protective measures may possibly reverse the path to cell death. In this context, it may be speculated that a secretion of SELENBP1 caused an associated decline of intracellular Se stores, thereby depriving the cardiomyocyte of the trace element with essential importance for antioxidative defense systems [37]. This notion is supported by clinical studies, where supplementation with Se along with coenzyme Q10 significantly reduced cardiovascular mortality in a prospective randomized double-blind controlled intervention study in European seniors [38].

There was an unexpected small difference between male and female patients concerning the velocity of the increase. However, peak values were similar and larger studies are needed in order to elucidate whether SELENBP1 secretion into the circulation constitutes another example of sex-specific differences in Se metabolism and selenoprotein expression [5]. The initial symptoms, disease development and mortality risk upon myocardial infarction show strong sex-specific differences that attenuate with age, highlighting sex as an important modifier of cardiovascular physiology and heart disease course [39]. It will therefore be important to conduct larger studies with both male and female patients in order to elucidate the importance of sex and age for the increase of circulating SELENBP1 concentrations, its dynamics and predictive value.

A limitation of this clinical study constitutes the relatively small sample size, limiting the results to testing the major hypothesis without providing the power for detailed and stratified analyses of subgroups of patients or sex-specific differences. A major strength of this study was the reliable, sensitive and highly reproducible analytical technique used, i.e., the novel luminometric immunoassay used for SELENBP1 quantification, which is based on specific monoclonal antibodies and is therefore available for verification analyses and additional clinical studies.

## 5. Conclusions

In this study, the kinetics and modifying factors of circulating SELENBP1 in response to myocardial injury were characterized. Increased concentrations reflected the duration of ischemia and myocardial damage, and reliably identified patients at risk of adverse outcomes. It is hoped that these insights will be useful for improving the care of cardiac surgery patients and help to identifying individuals with high risks for adverse events in order to raise increased attention and enable fast curative measures when first signs of worsening of the clinical condition become apparent.

## Figures and Tables

**Table 1:** Clinical characteristics of patients in relation to serum selenium-binding protein (1SELENBP1) concentrations.

Parameter	Total (*n *= 66)	SELENBP1-Positive * (*n *= 18)	SELENBP1-Negative (*n* = 48)
Age (years)	65 (58–75)	65 (60–75)	66 (56–75)
Female sex (*n*)	23% (15)	8% (5)	15% (10)
Male sex (n)	77% (51)	20% (13)	58% (38)
BMI (kg/m^2^)	27 (25–31)	27 (25–32)	28 (25–30)
EUROscore ^1^	4 (2–6)	6 (5–8)	3 (1–6)
Duration of intervention (min)	271 (207–330)	282 (225–317)	265 (200–331)
Duration of CPB (min)	120 (85–149)	148 (126–176)	100 (83–134)
Duration of ischemia (min)	72 (58–107)	112 (93–134)	63 (58–84)
Stay in hospital (days)	9 (7–11)	11 (8–14)	9 (7–10)
Bradycardia (n)	6% (4)	5% (3)	2% (1)
Exitus letalis (n)	5% (3)	5% (3)	0% (0)
Pneumonia (n)	5% (3)	5% (3)	0% (0)
Acute kidney failure (n)	5% (3)	3% (2)	2% (1)
Wound infection (n)	2% (1)	2% (1)	0% (0)
Cerebral ischemia (n)	2% (1)	2% (1)	0% (0)

Data are expressed as % (n) or median (IQR); * SELENBP1 positive; serum SELENBP1 concentration > 0.96 nM at 1 h after arrival on the ICU; ^1^ EURO score; system to assess early mortality after cardiac surgery, according to Nashed et al. [33].

**Table 2:** Association of elevated serum SELENBP1 concentrations with adverse outcomes.

Parameter	Threshold [nM]	OR	CI	*p*	PPV	NPV	AUC	*n*
15 min after CPB								
combined endpoint 1 *	0.988	22	3–194	0.0005	0.333	0.978	0.84	8
combined endpoint 2 *	0.820	18	2–152	0.0011	0.310	0.976	0.81	9
1 h ICU								
death	2.238	124	6.2777	0.0042	0.667	0.984	0.92	2
acute kidney injury	0.889	20	1–411	0.029	0.158	1.000	0.83	4
combined endpoint 1 *	0.956	30	3–269	0.0003	0.389	0.979	0.84	7
combined endpoint 2 *	0.889	33	4–296	<0.0001	0.421	0.979	0.84	7
6 h ICU								
death	1.292	63	4–1018	0.0077	0.500	0.984	0.88	3
maximal levels								
bradycardia	0.988	23	1–439	0.014	0.167	1.000	0.81	5
cerebral ischemia	1.396	25	1–544	0.034	0.143	1.000	0.90	3
combined endpoint 1 *	0.988	25	3–215	0.0003	0.333	0.980	0.88	8
combined endpoint 2 *	0.968	24	3–199	0.0003	0.333	0.979	0.86	9
stay in ICU for >40 day	0.870	4	1–10	0.013	0.484	0.795	0.70	14

* Combined endpoints; combined endpoint 1 comprises bradycardia, death, and cerebral ischemia; combined endpoint 2 comprises bradycardia, death, cerebral ischemia, as well as acute kidney injury. OR; odds ratio; CI, confidence interval; PPV, positive predictive value; NPV, negative predictive value, AUC, area under the curve.

## References

[1] Hondal R.J., Marino S.M., Gladyshev V.N. (2013). Selenocysteine in thiol/disulfide-like exchange reactions. Antioxid. Redox Signal..

[2] Brigelius-Flohé R., Flohé L. (2017). Selenium and redox signaling. Arch. Biochem. Biophys..

[3] Méplan C. (2015). Selenium and chronic diseases: A nutritional genomics perspective. Nutrients.

[4] Burk R.F., Hill K.E. (2015). Regulation of Selenium Metabolism and Transport. Ann. Rev. Nutr..

[5] Schomburg L., Schweizer U. (2009). Hierarchical regulation of selenoprotein expression and sex-specific effects of selenium. Biochim. Biophys. Acta.

[6] Schomburg L., Riese C., Renko K., Schweizer U. (2007). Effect of age on sexually dimorphic selenoprotein expression in mice. Biol. Chem..

[7] Rayman M.P. (2008). Food-chain selenium and human health: Emphasis on intake. Br. J. Nutr..

[8] Labunskyy V.M., Hatfield D.L., Gladyshev V.N. (2014). Selenoproteins: Molecular pathways and physiological roles. Physiol. Rev..

[9] Bansal M.P., Oborn C.J., Danielson K.G., Medina D. (1989). Evidence for two selenium-binding proteins distinct from glutathione peroxidase in mouse liver. Carcinogenesis.

[10] Elhodaky M., Diamond A.M. (2018). Selenium-Binding Protein 1 in Human Health and Disease. Int. J. Mol. Sci..

[11] Chang P.W., Tsui S.K., Liew C., Lee C.C., Waye M.M., Fung K.P. (1997). Isolation, characterization, and chromosomal mapping of a novel cDNA clone encoding human selenium binding protein. J. Cell Biochem..

[12] Lanfear J., Fleming J., Walker M., Harrison P. (1993). Different patterns of regulation of the genes encoding the closely related 56 kDa selenium- and acetaminophen-binding proteins in normal tissues and during carcinogenesis. Carcinogenesis.

[13] Yang M., Sytkowski A.J. (1998). Differential expression and androgen regulation of the human selenium-binding protein gene hSP56 in prostate cancer cells. Cancer Res..

[14] Fang W., Goldberg M.L., Pohl N.M., Bi X., Tong C., Xiong B., Koh T.J., Diamond A.M., Yang W. (2010). Functional and physical interaction between the selenium-binding protein 1 (SBP1) and the glutathione peroxidase 1 selenoprotein. Carcinogenesis.

[15] Xia Y.J., Ma Y.Y., He X.J., Wang H.J., Ye Z.Y., Tao H.Q. (2011). Suppression of selenium-binding protein 1 in gastric cancer is associated with poor survival. Hum. Pathol..

[16] Zhang S., Li F., Younes M., Liu H., Chen C., Yao Q. (2013). Reduced selenium-binding protein 1 in breast cancer correlates with poor survival and resistance to the anti-proliferative effects of selenium. PLoS ONE.

[17] Ha Y.S., Lee G.T., Kim Y.H., Kwon S.Y., Choi S.H., Kim T.H., Kwon T.G., Yun S.J., Kim I.Y., Kim W.J. (2014). Decreased selenium-binding protein 1 mRNA expression is associated with poor prognosis in renal cell carcinoma. World J. Surg. Oncol..

[18] Burk R.F., Hill K.E., Motley A.K. (2001). Plasma selenium in specific and non-specific forms. BioFactors.

[19] Bierl C., Voetsch B., Jin R.C., Handy D.E., Loscalzo J. (2004). Determinants of human plasma glutathione peroxidase (GPx-3) expression. J. Biol. Chem..

[20] Burk R.F., Olson G.E., Winfrey V.P., Hill K.E., Yin D. (2011). Glutathione peroxidase-3 produced by the kidney binds to a population of basement membranes in the gastrointestinal tract and in other tissues. Am. J. Physiol. Gastrointest. Liver Physiol..

[21] Schomburg L., Schweizer U., Holtmann B., Flohé L., Sendtner M., Köhrle J. (2003). Gene disruption discloses role of selenoprotein P in selenium delivery to target tissues. Biochem. J..

[22] Hill K.E., Zhou J., McMahan W.J., Motley A.K., Atkins J.F., Gesteland R.F., Burk R.F. (2003). Deletion of selenoprotein P alters distribution of selenium in the mouse. J. Biol. Chem..

[23] Hollenbach B., Morgenthaler N.G., Struck J., Alonso C., Bergmann A., Köhrle J., Schomburg L. (2008). New assay for the measurement of selenoprotein P as a sepsis biomarker from serum. J. Trace Elem. Med. Biol. Organ Soc. Miner. Trace Elem. (GMS).

[24] Barrett C.W., Short S.P., Williams C.S. (2017). Selenoproteins and oxidative stress-induced inflammatory tumorigenesis in the gut. Cell. Mol. Life Sci. CMLS.

[25] Polyzos S.A., Kountouras J., Mavrouli M., Katsinelos P., Doulberis M., Gavana E., Duntas L. (2019). Selenoprotein P in Patients with Nonalcoholic Fatty Liver Disease. Exp. Clin. Endocrinol. Diabetes Off. J. Ger. Soc. Endocrinol. Ger. Diabetes Assoc..

[26] Meyer H.A., Endermann T., Stephan C., Stoedter M., Behrends T., Wolff I., Jung K., Schomburg L. (2012). Selenoprotein P status correlates to cancer-specific mortality in renal cancer patients. PLoS ONE.

[27] Forceville X., Mostert V., Pierantoni A., Vitoux D., Le Toumelin P., Plouvier E., Dehoux M., Thuillier F., Combes A. (2009). Selenoprotein P, rather than glutathione peroxidase, as a potential marker of septic shock and related syndromes. Eur. Surg. Res. Eur. Chir. Forsch. Rech. Chir. Eur..

[28] Braunstein M., Kusmenkov T., Zuck C., Angstwurm M., Becker N.P., Bocker W., Schomburg L., Bogner-Flatz V. (2019). Selenium and Selenoprotein P Deficiency Correlates with Complications and Adverse Outcome After Major Trauma. Shock (AugustaGa).

[29] Kim K.S., Yang H.Y., Song H., Kang Y.R., Kwon J., An J., Son J.Y., Kwack S.J., Kim Y.M., Bae O.N. (2017). Identification of a sensitive urinary biomarker, selenium-binding protein 1, for early detection of acute kidney injury. J. Toxicol. Environ. Health Part A.

[30] Kühn E.C., Slagman A., Kühn-Heid E.C.D., Seelig J., Schwiebert C., Minich W.B., Stoppe C., Möckel M., Schomburg L. (2019). Circulating levels of selenium-binding protein 1 (SELENBP1) are associated with risk for major adverse cardiac events and death. J. Trace Elem. Med. Biol. Organ Soc. Miner. Trace Elem. (GMS).

[31] Suadicani P., Hein H.O., Gyntelberg F. (1992). Serum selenium concentration and risk of ischaemic heart disease in a prospective cohort study of 3000 males. Atherosclerosis.

[32] Wendt S., Schomburg L., Manzanares W., Stoppe C. (2019). Selenium in Cardiac Surgery. Nutr. Clin. Pract. Off. Publ. Am. Soc. Parenter. Enter. Nutr..

[33] Nashef S.A., Roques F., Michel P., Gauducheau E., Lemeshow S., Salamon R. (1999). European system for cardiac operative risk evaluation (EuroSCORE). Eur. J. Cardio Thorac. Surg..

[34] Ying Q., Ansong E., Diamond A.M., Yang W. (2015). A Critical Role for Cysteine 57 in the Biological Functions of Selenium Binding Protein-1. Int. J. Mol. Sci..

[35] Graham R.M., Frazier D.P., Thompson J.W., Haliko S., Li H., Wasserlauf B.J., Spiga M.G., Bishopric N.H., Webster K.A. (2004). A unique pathway of cardiac myocyte death caused by hypoxia-acidosis. J. Exp. Biol..

[36] Lee E.K., Shin Y.J., Park E.Y., Kim N.D., Moon A., Kwack S.J., Son J.Y., Kacew S., Lee B.M., Bae O.N. (2017). Selenium-binding protein 1: A sensitive urinary biomarker to detect heavy metal-induced nephrotoxicity. Arch. Toxicol..

[37] Boucher F.R., Jouan M.G., Moro C., Rakotovao A.N., Tanguy S., de Leiris J. (2008). Does selenium exert cardioprotective effects against oxidative stress in myocardial ischemia?. Acta Physiol. Hung..

[38] Alehagen U., Aaseth J., Johansson P. (2015). Reduced Cardiovascular Mortality 10 Years after Supplementation with Selenium and Coenzyme Q10 for Four Years: Follow-Up Results of a Prospective Randomized Double-Blind Placebo-Controlled Trial in Elderly Citizens. PLoS ONE.

[39] Canto J.G., Rogers W.J., Goldberg R.J., Peterson E.D., Wenger N.K., Vaccarino V., Kiefe C.I., Frederick P.D., Sopko G., Zheng Z.J. (2012). Association of age and sex with myocardial infarction symptom presentation and in-hospital mortality. JAMA.

